# Paclitaxel-induced scleroderma: A scoping review of reported cases with an illustrative case from our center

**DOI:** 10.1515/rir-2026-0034

**Published:** 2026-10-22

**Authors:** Milan Bogojevic, Rifat Medjedovic, Nevenka Lukovac Janjic, Ivana Vukovic, Milica Markovic, Marco Matucci-Cerinic

**Affiliations:** Department of Rheumatology, Clinical center of Montenegro, Podgorica, Montenegro; Institute of Oncology, Clinical center of Montenegro, Podgorica, Montenegro; Unit of Immunology, Rheumatology, Allergy and Rare Diseases (UnIRAR), IRCCS San Raffaele Scientific Institute, Vita-Salute San Raffaele University, Milan, Italy

**Keywords:** paclitaxel, taxanes, scleroderma, drug-induced fibrosis, autoantibodies

## Abstract

Paclitaxel-induced scleroderma is a rare but increasingly recognized adverse effect of taxane chemotherapy. Most cases present with distal edema and cutaneous sclerosis, usually without disease-specific autoantibodies or visceral involvement. This study aimed to map all published cases of paclitaxel-induced scleroderma and present a unique case characterized by dual anticentromere (ACA) and anti-cyclic citrullinated peptide (ACPA) antibody positivity. A scoping review of English-language literature was performed in PubMed and Scopus up to May 2025 using the terms “paclitaxel” OR “taxane” AND “scleroderma” OR “systemic sclerosis. ” Only human case reports or case series describing paclitaxel- or nab-paclitaxel–induced scleroderma were included. Data were extracted on demographics, cancer type, clinical features, serology, and outcomes, and summarized descriptively. Nineteen publications describing 21 patients were analyzed. Most patients were female (85.7%), with a mean age of 58.1 years. Cutaneous involvement was predominant, affecting both upper and lower extremities in 85.7% of cases. Raynaud’s phenomenon occurred in 28.6%, and visceral involvement was uncommon (9.5% interstitial lung fibrosis; 9.5% scleroderma renal crisis). Autoantibody profiles were largely negative; antinuclear antibodies (ANA) positivity was seen in 33.3% and ACA in only one case. Nearly all patients improved after paclitaxel withdrawal, with corticosteroids and/or methotrexate used in refractory disease. The presented patient developed scleroderma-like changes with ACA and ACPA positivity—an unprecedented serologic finding—together with transient synovitis and Raynaud’s phenomenon, confirming an immune-mediated overlap phenotype. Paclitaxel-induced scleroderma typically manifests as a reversible cutaneous fibrosing reaction distinct from idiopathic systemic sclerosis. However, rare cases with autoimmune serology suggest underlying immune activation in genetically predisposed individuals. The coexistence of ACA and ACPA positivity in the current case broadens the immunologic spectrum of taxane-induced scleroderma and highlights the need for further mechanistic and immunogenetic studies.

## Introduction

Systemic sclerosis (SSc) is a chronic autoimmune disease characterized by fibrosis of the skin and internal organs, vascular abnormalities, and autoantibody production. Although SSc is typically idiopathic, a number of environmental and pharmacologic agents have been implicated in scleroderma-like syndromes.^[1]^ Among these, several cytotoxic and immunomodulatory drugs used in oncology—such as bleomycin, gemcitabine, taxanes, and immune checkpoint inhibitors—have been associated with cutaneous and, less commonly, systemic fibrosing reactions. With the increasing use of these therapies, recognition of drug-related scleroderma has become clinically relevant to avoid irreversible fibrosis and to distinguish it from idiopathic SSc.^[2, 3, 4, 5, 6]^

Taxanes are a class of cytotoxic chemotherapeutic agents that stabilize microtubules, thereby inhibiting cell division and inducing apoptosis in rapidly proliferating cells. The two most widely used members of this class are paclitaxel and docetaxel, which are key components in the treatment of various solid tumors, including breast, ovarian, lung, and prostate cancers.^[7]^ Common adverse effects of taxanes include alopecia, myelosuppression, peripheral neuropathy, arthralgia and myalgia, hypersensitivity reactions, and nail or skin changes.^[8,9]^ Rare but notable immune-mediated toxicities, such as cutaneous sclerosis and systemic sclerosis-like syndromes, have also been reported, with mechanisms thought to involve immune dysregulation, endothelial injury, and cytokine-mediated fibroblast activation.^[10]^ To date, few cases of palcitaxel induced SSC were described but no prior case has shown concomitant anticentromere antibodies (ACA) and anticitrullinated protein antibodies (ACPA) positivity. We describe such a case and review the literature limited to paclitaxel-associated scleroderma in order to map existing evidence on paclitaxel-induced scleroderma, summarize reported clinical patterns, and identify knowledge gaps.

## Methods

A structured literature search was conducted in PubMed and Scopus up to May 2025 using the following terms (“paclitaxel” OR “taxane”) AND (“scleroderma” OR “systemic sclerosis”). Inclusion criteria were human case reports or series describing scleroderma or scleroderma-like skin changes induced by paclitaxel or nab-paclitaxel and papers published in English-language publications. Exclusion Criteria reports on non-taxane drugs (*e.g*., docetaxel, gemcitabine, bleomycin), reviews without new patient data and animal or *in vitro* studies.

From each eligible publication, the following data were extracted: year, cancer type, skin changes, presence of Raynaud’s phenomenon, autoantibodies, capillaroscopy findings, visceral involvement, treatment, and outcome.

Data were summarized descriptively and presented in tabular form. No formal risk-of-bias assessment was performed, consistent with scoping review methodology.

Study Selection Summary After screening titles and abstracts 27 publications reporting 36 cases of taxane-induced scleroderma were identified. After excluding eight reports involving patients treated with docetaxel,^[11, 12, 13, 14, 15, 16, 17, 18]^ data from 19 publications describing 21 patients who received paclitaxel or nab-paclitaxel were closely analyzed.^[2,10,19, 20, 21, 22, 23, 24, 25, 26, 27, 28, 29, 30, 31, 32, 33, 34, 35]^ In one of the 19 publication presenting 5 cases 4 patients were excluded, 3 because they were on docetaxel and 1 because there were no information about presenting cases.^[31]^

## Results

A structured summary of data from 19 publications describing 21 patients on paclitaxel induced scleroderma is provided in Table 1 to facilitate recognition of common clinical patterns.

**Table 1:** Reported cases of pacitaxel-induced scleroderma and present case

Author	Drug and dose	Cancer type	Clinical features beside scleroderma	Autoantibodies	Capillaroscopy	Outcome
Verhulis *et al*.^[19]^	Nabpaclitaxel 125 mg/m^2^	Pancreas	-	negative	normal	Improvment recived steroid ointment
Ketpueak *et al*.^[20]^	Paclitaxel 175 mg/m^2^	Ureter	RP, NSIP, Possible SRC	ANA +	normal	Died due suspected scleroderma renal crisis
Cury-Martins *et al*. 2 cases^[21]^ x	Paclitaxel N/A dose	Breast	-	Negative	Normal	Died before treatment
	Nabpaclitaxel N/A dose	Breast	-	Anti Ro +	Not preformed	Improved after withdrawal
Motegi *et al*.^[22]^	Nabpaclitaxel N/A dose	Pancreas	-	-	normal	Improved after withdrawal
Tani *et al*.^[23]^	Paclitaxel 80 mg/m^2^	Breast	-	ANA +	Normal	Improved after withdrawal
Shibao *et al*.^[24]^	Nabpacitaxel 125 mg/m^2^	Breast	pericardial effusion and pleural effusion. suspected SRC	ANA +	Normal	Died to kindney failiere
Winkelmann *et al*.^[10]^	Paclitaxel N/A dose	Ovarian	RP, esophageal dysmotility, myositis, digital pitting and telangiectasia	Negative	Dilatated capillaries	Improved receiving MTX plus steroids per os 20
Ogawa *et al*.^[25]^	Paclitaxel 175 mg/m^2^	Ovarian	RP, digital gangrene	ANA +	Normal	Improved scleroderma on Alprostadil but fatal outcome due carcinomatosis
Ohashi *et al*.^[26]^	Paclitaxel 80 mg/m^2^	Breast	-	Negative	Normal	Improvment after withdrawal, plus steroids per os and PUVA
Colson *et al*. 2 cases^[34]^	Paclitaxel N/A dose	Breast	-	Negative	Normal	Improved on Mtx and steroids per os
	Paclitaxel N/A dose	Breast	RP, dysphagia	negative	Normal	Improved on Mtx and steroids per os
Takahashi *et al*.^[2]^	Paclitaxel 100 mg/m^2^	Breast	-	Negative	Normal	Improved after withdrawal, on steriods
Konishi *et al*.^[27]^	Paclitaxel 175 mg/m^2^	Ovarian	-	ANA	Normal	Progresion on steroid ointment and withdrawal
Pedersen *et al*.^[28]^	Paclitaxel 175 mg/m^2^	Ovarian	RP, athralgia, microstomia and dyspnoea, lower DLCO	Negative	Normal	Treatment consisted of PUVA and methotrexate no improvment
Kawakami *et al*.^[30]^	Paclitaxel N/A dose	Breast	RP	ANA + ACA +	Normal	Improved after withdrawal
Itoh *et al*.^[31]^	Paclitaxel 80 mg/m^2^	Breast	-	Negative	normal	Improved after withdrawal plus oral steroids
Kupfer *et al*.^[32]^	Paclitaxel 175 mg/m^2^	Peritoneal	-	Negative	Not preformed	Improved after withdrawal
Farrant *et al*.^[35]^	Paclitaxel N/A dose	Ovarian	-	Negative	normal	No improvement after withdrawal
De Angelis *et al*.^[29]^	Paclitaxel 175 mg/m^2^	Ovarian	esophageal dysmotility, telangiectasia	ANA	Active pattern	Improvment with oral and d-penicillamine
Lauchi *et al*.^[33]^	Paclitaxel 175 mg/m^2^	Peritoneal	Microstomia, Calcinosis, Pulmonary fibrosis	Negative	Normal	Improvement on iloprost and physiotherapy
Our case	Paclitaxel 175 mg/m^2^	Ovarian	RP, telangiectasia, synovitis	ANA, ACA, anti ACPA	Non specific	Improved on steroids and HCQ

RP, Raynaud’s Phenomenon; NSIP, Non-specific interstitial pneumonia; SRC, Scleroderma renal crisis; DLCO, Diffusing capacity of the lungs for carbon monoxide; ANA, antinuclear antibodies; ACA, anticentromere antibodies; ACPA, Anti-citrullinated protein antibodies; MTX, methotrexate; PUVA, photochemotherapy; HCQ, Hydroxychlo-roquine; N/A, not available.

A total of 21 patients with paclitaxel-induced scleroderma were identified from 19 publications. Among them, 3 were male (14.3%)^[19,21,22]^ and 18 female (85.7%). The mean age at onset was 58.1 years (SD 9.99; range 37–71). Four patients (19%) received nab-paclitaxel,^[19,21,22,24]^ while the remaining 17 (85.7%) were treated with conventional paclitaxel.

Regarding cutaneous distribution, 18 patients (85.7%) exhibited sclerodermoid changes involving both upper (UE) and lower extremities (LE). In 8 patients (38.1%), symptoms began in the LE and later extended to the UE, while in 3 patients (14.3%) the process started in the UE and progressed to the LE. In 7 cases (33.3%), both regions were affected simultaneously. Three patients (14.3%) had skin involvement limited to the UE only, and three (14.3%) also developed facial sclerosis.

Concerning the underlying malignancy, 10 patients (47.6%) were treated for breast cancer,^[2,21,23,24,26,30,31,34]^ 6 (28.6%) for ovarian cancer,^[10,25,27, 28, 29,35]^ 2 (9.5%) for peritoneal carcinoma,^[32,33]^ 2 (9.5%) for pancreatic cancer,^[19,22]^ and 1 (4.8%) for ureteral carcinoma.^[20]^

The onset of scleroderma-like symptoms ranged from 10 days after the first chemotherapy cycle^[32]^ to 60 months after the fifth,^[35]^ though the majority (14 patients; 66.7%) developed symptoms after the fourth cycle. The administered dose ranged from 80 mg/m^2^ to 175 mg/m^2^. Based on the available data, we did not observe any association between the drug dose and the time of onset of scleroderma-like symptoms.

Cutaneous manifestations were the most frequent, whereas visceral involvement was rare, with two patients (9.5%) presenting interstitial lung fibrosis,^[20,33]^ one (4.8%) with myositis,^[10]^ two (9.5%) with esophageal dysmotility,^[10,29]^ one (4.8%) with pleural and pericardial effusion,^[24]^ and two (9.5%) with possible scleroderma renal crisis.^[20,24]^ Synovitis was not observed in any of the paclitaxel cases, although one docetaxel-treated patient presented with synovitis and was ACA-positive.^[13]^ Raynaud’s phenomenon occurred in 6 out of 21 patients (28.6%),^[10,20,25,28,30,34]^ capillaroscopy showed one active pattern^[29]^ and dilateted capillares in one.^[10]^

Serologically, the majority of cases were autoantibody-negative. Seven patients (33.3%) had ANA positivity,^[20,23, 24, 25,27,29,30]^ with ACA detected in one (4.8%)^[30]^ and low anti-Ro antibody titers in one (4.8%).^[21]^

Regarding outcomes, four patients (19.0%) died—one due to a suspected scleroderma renal crisis,^[20]^ one due to worsening renal function (24), and two from progression of the underlying malignancy.^[21,25]^ All patients discontinued paclitaxel upon diagnosis; 15 (71.4%) showed clinical improvement, while 3 (14.3%) experienced no improvement.^[27,28,35]^ Seven patients (33.3%) received corticosteroids, and four (19.0%) were treated with methotrexate as part of immunosuppressive management.

## Case Presentation

A 64-year-old woman was diagnosed in 2020 with high-grade serous ovarian adenocarcinoma, BRCA1/2 negative, with peritoneal metastases. She was started on combination chemotherapy with carboplatin (CBDCA) and paclitaxel (Taxol) every three weeks, together with bevacizumab. A good therapeutic response was achieved, with marked disease regression and improvement in performance status. After six cycles, treatment with paclitaxel was discontinued due to grade 3 polyneuropathy, and maintenance therapy with bevacizumab and femosol was continued until October 2021, after which she remained on femosol monotherapy with stable disease. In 2022, progressive disease (PD) was documented with lymphatic and peritoneal dissemination. She was re-treated with carboplatin plus gemcitabine and bevacizumab, achieving a complete remission after six cycles. Maintenance bevacizumab was continued until April 2023. In August 2023, MRI of the abdomen revealed disease progression with intra- and retroperitoneal lymphatic masses and ascites. A platinum-based re-treatment (cisplatin) was attempted, but after four cycles further progression occurred. In January 2024, paclitaxel was reintroduced in the platinum-resistant phase. During the new course of treatment, she developed carpal tunnel syndrome, and by March 2024 (after eight cycles) she presented with bilateral hand edema. She was referred to rheumatology for evaluation.

At the rheumatologic assessment, she reported that at the time her hands became swollen, she also experienced Raynaud’s phenomenon and inflammatory joint pain. She denied dryness of eyes or mouth, dysphagia, or dyspnea. Physical examination revealed puffy fingers with sclerodactyly, synovitis of the radiocarpal and third metacarpophalangeal joints bilaterally with telangiectasias and no digital ulcers.

Investigations included musculoskeletal ultrasound of the hands, nailfold capillaroscopy, X-rays of hands, feet, and chest, Doppler sonography of hands and echocardiography. Serologic tests comprised rheumatoid factor (RF), ACPA, ANA, ACA, anti ds DNA, ENA panel (anti-Scl-70, RNP, Ro, La), complement (C3, C4), and thyroid function (TSH, fT3, fT4).

Immunological findings showed ANA positive (1:160 speckled pattern), ACA positive, and ACPA positive. Anti ds DNA and ENA panel were negative. Ultrasound revealed distension of the radiocarpal joint capsule bilaterally (grade II) without power Doppler signal and mild synovitis of MCP III joints (grade I–II). A distended tendon sheath of the fourth extensor compartment (grade II–III) was also noted, without Doppler signal (Figure 1). There was pronounced subcutaneous edema but no erosive changes. Chest X-ray and cardiac echo sonography were normal. Capillaroscopy demonstrated nonspecific abnormalities, including tortuous and dilated capillary loops with sluggish flow, but no megacapillaries or microhemorrhages. Chest X ray and spirometry were normal. Echocardiogram showed preserved cardiac function, Doppler sonography of hands was normal. Based on the temporal association with paclitaxel re-exposure and absence of visceral involvement, a diagnosis of paclitaxel-induced systemic sclerosis without organ involvement was made. Treatment included hydroxychloroquine (400 mg/day), prednisone (20 mg/day with tapering), and acetylsalicylic acid. The patient continued oncologic therapy under close rheumatologic monitoring.

**Figure 1:**
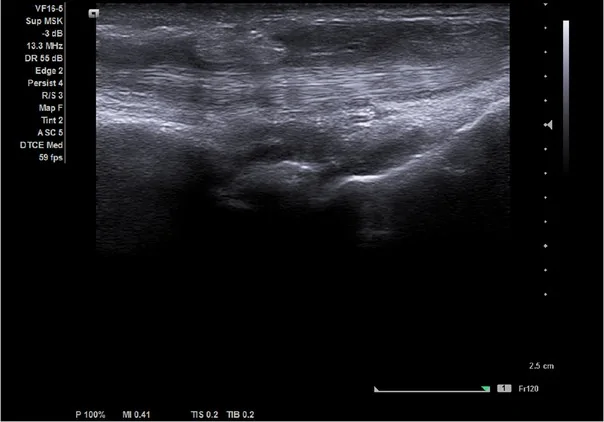
Ultrasound of the hand radiocarpal joint, grey scale, longitudinal. Anehoic zone in the area of the radiocarpal joint with associated tenosynovitis of the extensor tendon

After initiation of therapy, the patient showed marked clinical improvement, with reduction in joint pain and hand edema. Follow-up ultrasound confirmed significant regression of synovial and tendon sheath distension and reduction of subcutaneous edema. In agreement with the oncology team, paclitaxel therapy was continued until a total of 14 cycles, after which it was discontinued due to progressive disease and switch to cyclophosphamide. After paclitaxel cessation, rheumatologic symptoms nearly completely resolved, consistent with drug-induced systemic sclerosis in remission.

## Discussion

Drug-induced scleroderma (DISc) represents a heterogeneous group of fibrosing conditions triggered by diverse pharmacologic exposures. Although various agents—including bleomycin, gemcitabine, and immune checkpoint inhibitors—have been implicated, taxanes remain the prototypical cause of chemotherapy-associated scleroderma-like syndromes.^[2, 3, 4, 5, 6]^ The findings of our scoping review, together with the present case, confirm a consistent clinical pattern of paclitaxel-induced sclerosis characterized by distal edema progressing to induration, female predominance, absence of classical SSc autoantibodies, and reversibility after drug withdrawal. Nevertheless, the immunologic spectrum may be broader than previously recognized, as illustrated by our patient, who exhibited both anticentromere (ACA) and anti-citrullinated peptide (ACPA) antibodies—an association not previously reported.

Taken together, the available literature suggests a relatively consistent clinical and biological pattern of taxane-induced scleroderma, although important variability remains in immunologic features, severity, and reversibility.

The precise pathophysiology of taxane-induced scleroderma remains incompletely understood. Several mechanisms have been proposed, including vascular injury, cytokine-mediated fibroblast activation, and immune dysregulation. Paclitaxel promotes microtubule stabilization and interferes with normal endothelial cell turnover, leading to endothelial apoptosis, microangiopathy, and tissue ischemia, which may subsequently trigger fibrotic remodeling.^[36]^ Experimental studies demonstrate that paclitaxel upregulates profibrotic cytokines such as TGF-β, IL-6, and TNF-α, and increases expression of connective tissue growth factor (CTGF), all of which drive fibroblast proliferation and collagen synthesis.^[37,38]^ However, these mechanisms remain largely speculative, as most data are derived from *in vitro* models rather than patient-based studies, highlighting a significant gap in translational understanding.

At the molecular level, Takahashi *et al*. described a distinct fibroblast activation pattern in taxane-induced sclerosis. Downregulation of the Fli-1 transcription factor in dermal fibroblasts—without the parallel endothelial suppression observed in idiopathic SSc—suggests a fibroblast-restricted profibrotic signal.^[2]^ Complementary histopathological studies have demonstrated deposition of glycosaminoglycans and versican, supporting the hypothesis that taxanes may disrupt extracellular matrix homeostasis through impaired degradation and increased matrix accumulation.^[39]^ Collectively, these findings suggest that paclitaxel may act both as a direct fibroblast stimulator and an indirect endothelial stressor, promoting fibrosis without the classic vasculopathy of idiopathic SSc.

Consistent with the literature, serologic profiles in most reported cases are negative for antinuclear and disease-specific antibodies, with only isolated cases showing ACA or anti-Ro positivity.^[21,30]^ Capillaroscopic data, when available, reveal either nonspecific or minimally active changes rather than the classic “scleroderma pattern, ” supporting the notion that vascular remodeling is secondary rather than primary in taxane-associated disease.^[10,29]^ These observations reinforce the concept that drug-induced sclerosis is mechanistically distinct from idiopathic SSc, representing a predominantly reactive, non-autoimmune fibrotic process in most patients.

Nevertheless, it has been proposed that chronic taxane exposure may unmask latent autoimmunity in genetically predisposed individuals, promoting autoreactive B-cell activation and epitope spreading.^[40]^ The coexistence of ACA and ACPA antibodies in our patient supports this hypothesis and suggests potential cross-activation of systemic sclerosis– and rheumatoid arthritis–related immune pathways.

Despite the growing number of case reports, significant knowledge gaps remain. No large-scale immunogenetic or transcriptomic studies have evaluated host susceptibility to taxane-induced fibrosis.^[40]^ The lack of standardized serologic and histopathologic characterization across reports limits comparability and hinders the development of clear diagnostic criteria.^[20]^ Furthermore, the relationship between cumulative dose, treatment duration, and fibrotic risk remains uncertain, and mechanistic insights are largely extrapolated from experimental models rather than patient-derived data.

Another unresolved issue, also highlighted by our case, concerns the reversibility of fibrosis. While most patients show substantial improvement following drug withdrawal and short-term corticosteroid therapy, others experience persistent sclerosis despite immunosuppression.^[27,35]^ Whether this variability reflects differences in host immune response, drug formulation (paclitaxel *vs*. nab-paclitaxel), or concomitant therapies remains unclear. Longitudinal studies with systematic follow-up are needed to better define outcomes and identify predictors of recovery.

The present case expands the phenotypic and serologic spectrum of paclitaxel-induced scleroderma in several ways. The coexistence of ACA and ACPA positivity has not been previously reported and suggests immune cross-reactivity between systemic sclerosis and rheumatoid arthritis pathways. The presence of Raynaud’s phenomenon and transient synovitis further supports an overlap phenotype rather than a purely toxic reaction. In addition, this case illustrates that clinical improvement may be achieved without complete discontinuation of paclitaxel when close multidisciplinary monitoring allows continuation of oncologic therapy under immunomodulatory treatment, with potential implications for balancing antitumor efficacy and immune toxicity.

Overall, the literature highlights several consistent themes. Paclitaxel-induced scleroderma differs from idiopathic systemic sclerosis in its clinical course, serologic profile, and reversibility. Fibroblast activation appears to be central, although immune-mediated mechanisms may contribute in selected cases. Current evidence is limited by small sample sizes, heterogeneity in reporting, and lack of standardized diagnostic criteria. Management remains largely empirical, with drug withdrawal and corticosteroids as the main strategies, while long-term outcomes remain insufficiently characterized.

Finally, the integration of our case with the available literature supports the concept that taxane-induced scleroderma represents a spectrum ranging from nonimmune fibroblast activation to immune-mediated fibrosis. Recognition of this continuum has important clinical implications for diagnosis, monitoring, and treatment. Further research integrating clinical, immunologic, and molecular data will be essential to better define susceptibility factors and optimize individualized management strategies.

## Conclusions

We describe the first known case of paclitaxel-induced scleroderma with dual ACA and ACPA antibody positivity. This report emphasizes the role of immune and genetic predisposition in the pathogenesis of drug-induced scleroderma and underscores the need for vigilance among oncologists and rheumatologists. Given the growing global burden of malignancy and the expanding use of cytotoxic and immunomodulatory agents, it is reasonable to expect a rising incidence of drug-associated scleroderma and related fibrosing disorders. Early identification and multidisciplinary collaboration between oncologists, dermatologists, and rheumatologists are essential to avoid irreversible tissue damage while maintaining antitumor efficacy. Early recognition and cessation of the offending agent can lead to improvement, while immunosuppressive therapy may be beneficial in persistent cases.
